# Association Between Exposure to Particulate Matter Air Pollution with Risk of Obesity Among Children and Adolescents in Northern and Central Taiwan

**DOI:** 10.3390/children11121545

**Published:** 2024-12-19

**Authors:** Shu-Wei Hu, Hueng-Chuen Fan, Chuan-Mu Chen

**Affiliations:** 1 Division of Pediatric Gastroenterology, Department of Pediatrics, Tungs’ Taichung MetroHarbor Hospital, Taichung 435403, Taiwan; husw0310@gmail.com; 2 Division of Adolescent Medicine, Department of Pediatrics, Tungs’ Taichung MetroHarbor Hospital, Taichung 435403, Taiwan; 3 Division of Pediatric Neurology, Department of Pediatrics, Tungs’ Taichung MetroHarbor Hospital, Taichung 435403, Taiwan; fanhuengchuen@yahoo.com.tw; 4 Department of Life Sciences, Doctoral Program in Translational Medicine, National Chung Hsing University, Taichung 402202, Taiwan; 5 Rong Hsing Research Center for Translational Medicine, National Chung Hsing University, Taichung 402202, Taiwan; 6 Department of Rehabilitation, Jen-Teh Junior College of Medicine, Nursing and Management, Miaoli 356006, Taiwan; 7 The iEGG and Animal Biotechnology Research Center, National Chung Hsing University, Taichung 402202, Taiwan; 8 Center for General Educational, National Quemoy University, Kinmen 892009, Taiwan

**Keywords:** obesity, children, adolescents, PM_2.5_, PM_10_

## Abstract

Introduction: The present study investigated the relationship between air pollution, specifically PM_2.5_ and PM_10_, and childhood and adolescent obesity in northern and central Taiwan. Previous research has shown a positive correlation between air pollution and pediatric obesity, but no study has been conducted in Taiwan. We used data from the K-12 Education Administration, Ministry of Education, and the Taiwan Air Quality Monitoring Network to analyze the association between PM_2.5_ and PM_10_ exposures and obesity rates among elementary and junior high school students. Methods: Data on students’ height and weight were combined with air pollution data obtained from monitoring stations to assess exposure. A multivariable model estimated the relative risk and 95% confidence intervals of obesity linked to PM_2.5_ and PM_10_ exposures. Cities were categorized into quartiles (Q1–Q4) based on pollutant accumulation to compare the obesity rates. Results: Students living in areas with higher PM_2.5_ and PM_10_ exposures (Q4) had a significantly higher risk of obesity than those living in areas with lower exposures (Q1). The effect was more pronounced in girls and older students, with PM_2.5_ exhibiting a stronger relationship than PM_10_. Conclusions: PM_2.5_ and PM_10_ exposures are significantly associated with an increased obesity risk in children and adolescents, particularly in girls and older students. Further research is needed to explore the underlying mechanisms and to control for socioeconomic and demographic factors.

## 1. Introduction

Air pollution exposure is associated with many health problems, including obesity, metabolic syndrome (MetS), attention-deficit/hyperactivity disorder (ADHD), allergic rhinitis, asthma, and cardiovascular disease [1,2,3,4,5,6,7,8]. There are many types of air pollutants, including nitrogen dioxide, sulfur dioxide, carbon monoxide, ozone, PM_2.5_, and PM_10_. PM_2.5_ and PM_10_ are particulate matters with a diameter of ≤2.5 mcg/m^3^ and ≤10 mcg/m^3^, respectively.

Pediatric obesity, which is a major health problem worldwide, has many comorbidities, including metabolic dysfunction-associated fatty liver disease, dyslipidemia, hypertension, diabetes mellitus, obstructive sleep apnea, psychosocial disturbances, and precocious puberty [9,10,11]. Recent studies have shown positive correlations between pediatric obesity and air pollution [1,12,13,14]. In addition, Sundram et al. found that PM_2.5_ exposure is associated with increased calorie intake and appetite [2]. However, the correlation between pediatric obesity and air pollution in Taiwan has not been studied yet. Given that most published studies on air pollution and pediatric obesity were conducted in developing countries, the socioeconomic status, food preference, and medical level may be different between these countries and R.O.C. (Taiwan), which is a developed country. This study could show whether the PM_2.5_ also had a positive correlation with pediatric obesity in Taiwan. Additionally, we also surveyed whether PM_10_ had the same effect as PM_2.5_ or not.

Most studies on obesity in Taiwan used data from the Taiwan’s National Health Insurance Research Database, but this method has bias, especially in pediatric obesity and overweight studies. The estimated prevalence rate may be underestimated.

We used a new method through school health examinations to make the real prevalence close. In our previous unpublished study, we found that MetS and fatty liver are common in adolescents with severe obesity. Therefore, we aimed to analyze the effect of PM_2.5_ and PM_10_ exposures on children and adolescent obesity in northern and central Taiwan. In addition, we also sought to investigate whether the severity of air pollution influences the development of pediatric obesity, so that we can limit the outdoor activities of children and adolescents when the air pollution level exceeds the standard threshold.

## 2. Materials and Methods

### 2.1. Data Source

In Taiwan, the body weight and height of every student in elementary and junior high schools were recorded during the annual school health examinations. The K-12 Education Administration, Ministry of Education, R.O.C. (Taiwan), saved the data and analyzed the percentage of students who were overweight or obese by sex for each grade level in each town or district. We assumed that, for this age group, the school was located near the area of residence. Given that the data were not published, we obtained the data after submitting an official request letter to the K-12 Education Administration. To determine the total number of students classified by sex for each town or district, we also used the data from the Ministry of Education, R.O.C. (Taiwan) [15]. Then, we estimated the numbers of obese students by sex in different towns or districts by combining the abovementioned data. We selected all the elementary and junior high schools from 23 regions [Shilin, Zhongzheng, Wanhua, Songshan, Zhongshan, Datong, Taoyuan, Dayuan, Zhongli, Pingzhen, Longtan, Guanyin, Fengyuan, Dali, North (Taichung), Xitun, Shalu, Changhua, Erlin, Xianxi, Nantou, Puli, Zhushan] according to the air quality-monitoring stations in northern and central Taiwan.

The Taiwan Air Quality Monitoring Network database contains data on the daily concentrations of nitrogen dioxide, sulfur dioxide, carbon monoxide, ozone, PM_2.5_, and PM_10_ since 2005, and this database is maintained by the Ministry of Environment, R.O.C. (Taiwan) [16]. The Taiwan Air Quality Monitoring Network includes 77 air quality-monitoring stations, which were established based on the population density in Taiwan. The data from this database were published. Then, we selected all the stations (*n* = 23) in northern and central Taiwan.

The two databases were merged according to the living area and the location of the air quality-monitoring stations. Each participant was assigned to the groups stratified according to the pollutant exposure concentration of the area based on the data collected from the monitoring station located in their area of residence.

The present study was approved by the Institutional Review Board of the Research Ethics Committee of the Tung’ Taichung MetroHarbor Hospital (112044).

### 2.2. Study Population, Outcome, and Comorbidities

We enrolled grade 1–6 elementary students and grade 1–3 junior high school students who lived in areas with air quality-monitoring stations in northern (12 stations) or central Taiwan (11 stations). Obesity was defined as a body mass index (BMI) above the 95th percentile for age and sex, and overweight was defined as a BMI above the 85th percentile for age and sex [17].

### 2.3. Statistical Analysis

A multivariable model was used to estimate the relative ratio (RR) and 95% confidence interval (CI) of the association between the risk of obesity and PM_2.5_ or PM_10_ exposure.

**Figure 1:**
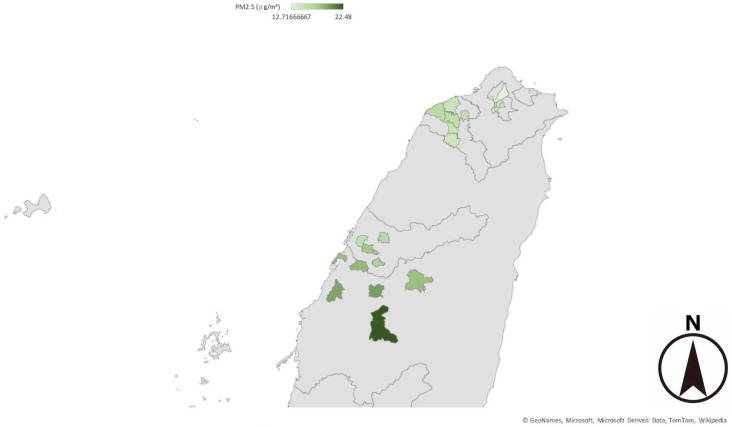
Daily average PM_2.5_ concentration obtained by the monitoring stations during 2017–2022. The values were higher in central Taiwan than in northern Taiwan. In central Taiwan, the values were higher in the mountain-side area than the seaside area.

**Figure 2:**
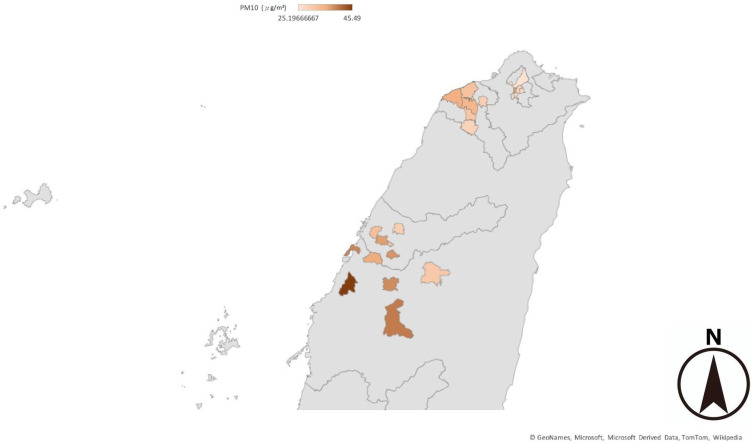
Daily average PM_10_ concentration obtained by the monitoring stations during 2017–2022. The values were higher in central Taiwan than in northern Taiwan. In central Taiwan, the values were higher in the seaside area than in the mountain-side area.

We arranged the cities in the order of PM_2.5_ accumulation concentration from 2017 to 2022 (Figure 1 and Figure 2) and categorized the cities into the following four quartiles: Q1, Q2, Q3, and Q4, with the first quartile (Q1) containing cities in the lowest 25% quartile of PM_2.5_ accumulation concentration and the fourth quartile (Q4) containing cities in the highest 25% quartile of PM_2.5_ accumulation concentration (Table 1 and Table 2). Then, we analyzed the HR, OR, and CI and compared the values between the elementary school or junior high school students with obesity in the year 2022 in Q1 and those in Q4. Moreover, the same analysis was carried out between the students in Q3 and those in Q1 and between those in Q2 and in Q1. We also used the same method to analyze PM_10_.

In addition, we analyzed the correlation coefficient (R) between the annual PM_2.5_ or PM_10_ concentration and the percentage of boys who were overweight, boys who were obese, girls who were overweight, and girls who were obese in the elementary and junior high schools. Given that the PM_2.5_ and PM_10_ levels have high fluctuations in the same year, we analyzed the average of the top 10% level in the year to know if the correlation between obesity and the peak level of air pollution was higher than the annual average level.

All the statistical analyses were performed using SPSS software (version 20, IBM Corp., Armonk, NY, USA). Statistical significance was set at 0.05. A high correlation was defined as the R between 0.6 and 0.9.

## 3. Results

Altogether, 206,758 (male: 106,586, female: 100,172) elementary school studies and 99,361 (male: 50,387, female: 48,974) junior high school students in 2022 were enrolled in this study.

In the PM_2.5_ accumulation concentration analysis, the Q1 cities were Shilin, Zhongzheng, Wanhua, Songshan, Zhongshan, and Longtan; the Q2 cities were Dayuan, Pingzhen, Taoyuan, Datong, Shalu, and Zhongli; the Q3 cities were Guanyin, Fengyuan, Dali, North (Taichung) and Xitun; and the Q4 cities were Puli, Xianxi, Changhua, Erlin, Nantou, and Zhushan. Q1 involved 3687.77 boys (13.1%) and 2129.72 girls (8.1%) in elementary schools, and 2027.03 boys (17.4%) and 1050.3 girls (9.3%) with obesity in junior high schools. Q2 involved 7172.61 boys (16.3%) and 4311.19 girls (10.4%) in elementary schools, and 4797.1 boys (21.3%) and 2527.27 girls (12.2%) with obesity in junior high schools. Q3 involved 3199.54 boys (14.9%) and 2083.62 girls (10.1%) with obesity in elementary schools, and 2013.87 boys (20.2%) and 1386.68 girls (12.2%) with obesity in junior high schools. Q4 involved 2424.34 boys (18.8%) and 1531.49 girls (12.9%) with obesity in elementary schools, and 1517.38 boys (24.1%) and 850.16 girls (15.0%) with obesity in junior high schools. The boys or girls in the elementary or junior high schools in Q4 had the highest RRs as compared to those in Q2 and Q3. The RRs of the girls and boys in the junior high schools in Q4 were 1.604 (95% CI: 1.474–1.745, *p* < 0.001) and 1.382 (95% CI: 1.303–1.466, *p* < 0.001), respectively. The RRs of the girls and boys in the elementary schools in Q4 were 1.582 (95% CI: 1.487–1.683, *p* < 0.001) and 1.435 (95% CI: 1.369–1.504, *p* < 0.001), respectively (Table 3 and Table 4).

In the PM_10_ accumulation concentration analysis, the Q1 cities were Zhongzheng, Shilin, Wanhua, Songshan, Zhongshan, and Longtan; the Q2 cities were Taoyuan, Fengyuan, Puli, North (Taichung), Pingzhen, and Dayuan; the Q3 cities were Shalu, Zhongli, Guanyin, Changhua, and Xitun; and the Q4 cities were Datong, Dali, Nantou, Xianxi, Zhushan, and Erlin. Q1 involved 3687.77 boys (13.1%) and 2129.72 girls (8.1%) with obesity in elementary schools, and 2027.03 boys (17.4%) and 1050.3 girls (9.3%) with obesity in junior high schools. Q2 involved 5477.34 boys (15.9%) and 3438.55 girls (10.5%) with obesity in elementary schools, and 3904.32 boys (21.2%) and 2231.15 girls (11.9%) with obesity in junior high schools. Q3 involved 5209.69 boys (16.0%) and 3178.88 girls (10.4%) with obesity in elementary schools, and 2706.8 boys (21.1%) and 1470.28 girls (12.8%) with obesity in junior high schools. Q4 involved 2024.49 boys (17.9%) and 1220.41 girls (11.5%) with obesity in elementary schools, and 1709.77 boys (22.9%) and 1038.26 girls (13.9%) with obesity in junior high schools. The boys or girls in the elementary or junior high schools in Q4 had the highest RRs compared those in Q2 and Q3. The RRs of the girls and boys in the junior high schools in Q4 were 1.488 (95% CI: 1.373–1.613, *p* < 0.001) and 1.316 (95% CI: 1.243–1.394, *p* < 0.001), respectively. The RRs of the girls and boys in the elementary schools in Q4 were 1.414 (95% CI: 1.323–1.511, *p* < 0.01) and 1.365 (95% CI: 1.299–1.434, *p* < 0.001), respectively (Table 5 and Table 6).

The highest RR of obesity to PM_2.5_ was 1.604 in the junior high school girls in Q4–Q1. Meanwhile, the RR of obesity to PM_2.5_ was 1.382 in junior high school boys in Q4–Q1. The highest RR of obesity to PM_10_ was 1.488 in the junior high school girls in Q4–Q1. Concurrently, the RR of obesity to PM_10_ was 1.316 in the junior high school boys in Q4–Q1. The results indicated that girls had a stronger relationship between obesity and both PM_2.5_ and PM_10_ exposures as compared to boys. In addition, PM_2.5_ had a greater influence in obesity, as compared to PM_10_.

The nationwide percentages of boys and girls who were overweight in elementary schools in 2020, 2021, and 2022 were 13.56%, 14.38%, and 13.81%, respectively, and 11.02%, 11.27%, and 10.84%, respectively. The nationwide percentages of boys and girls who were obese in elementary schools in 2020, 2021, and 2022 were 15.34%, 16.85%, and 16.64%, respectively, and 10.79%, 11.38%, and 11.12%, respectively.

The nationwide percentages of boys and girls who were overweight in junior high schools in 2020, 2021, and 2022 were 13.34%, 13.81%, and 13.26%, respectively, and 11.77%, 11.96%, and 11.39%, respectively. The nationwide percentages of boys and girls who were obese in junior high schools in 2020, 2021, and 2022 were 20.90%, 22.63%, and 21.94%, respectively, and 13.34%, 13.56%, and 13.26%, respectively.

The PM_2.5_ annual average, PM_2.5_ annual top 10% value average, PM_10_ annual average, and PM_10_ annual top 10% value average from 2020 to 2022 were analyzed (Table 7 and Table 8). In 2020, the PM_2.5_ annual average was not highly correlated to overweight or obesity. However, the PM_2.5_ annual top 10% average showed a high correlation with obesity in boys in elementary schools (R: 0.68), girls in elementary schools (R: 0.61), and boys in junior high schools (R: 0.64). The PM_10_ annual average showed a high correlation with obesity in boys in junior high schools (R: 0.65). The PM_10_ annual top 10% average showed a high correlation with obesity in boys in elementary (R: 0.60) and junior high schools (R: 0.70). Similar findings were also found in 2021 and 2022, and the top 10% values of PM_2.5_ and PM_10_ showed higher correlations to obesity but not to overweight as compared to the annual average (Table 9).

## 4. Discussion

In the present study, the air pollution data showed that the general air condition was better in northern Taiwan than in central Taiwan during the period from 2017 to 2022. The condition improved gradually in 6 years, and we assumed that it was attributed to the air pollution policy and coronavirus disease 2019 (COVID-19) pandemic, as the industrial activity was restricted during the pandemic.

Some studies have shown that air pollution has a positive effect on obesity [1,12,14,18,19,20]. Tamayo-Ortiz et al. reported that a 10-mcg/m^3^ increase in PM_2.5_ was associated with odds ratios (ORs) of 3.53 and 3.79 in 2006 and 2012, respectively, among adolescents in Mexico [1]. Parasin’s team found PM_2.5_ was associated with a significantly increased risk (6%) of childhood obesity in their meta-analysis study [12]. Zheng et al. reported that exposure to PM_2.5_, PM_10_, and NO_X_ increased the risk of overweight or obesity, especially in Asia [14]. The air pollution also had a positive correlation with MetS in children and adolescents. Zhang et al. showed that the ORs of MetS in children and adolescents associated with a 10-mcg/m^3^ increase in PM_2.5_ and PM_10_ were 1.31 and 1.32, respectively [8]. Huang et al. also found that a 10-mcg/m^3^ increment of PM_2.5_ was associated with a weight gain of 0.421 kg/m^2^, increased BMI of 13.5%, and increased overweight/obesity risk among Chinese adults [20].

Chen et al. reported that long-term PM_2.5_ exposure in northern Taiwan is associated with decreased skeletal muscle mass and increased body fat mass. Changes in the body composition for every 1.41-mcg/m^3^ increase in PM_2.5_ concentration included a 0.4 kg (2.0%) decrease in skeletal muscle mass and a 0.7 kg (3.6%) increase in body fat mass [18]. This study demonstrated that the exposure to PM_2.5_ indeed influenced the change in body composition, but this previous only analyzed the data of people aged >65 years. It remains unclear whether this effect can also be seen in pediatric patients in Taiwan.

The Tong et al. and Wang et al. studied the impact of PM_2.5_ and childhood obesity in China [21,22]. Tong et al. enrolled 4284 children aged 6–8 years included in the Chongqing Children Health Cohort in 2014–2015, and these children were followed up in 2019. For every 5-mcg/m^3^ increase in PM_2.5_ levels, the risk of central obesity was increased by 1.26 times [21]. Wang et al. conducted a longitudinal study among Chinese children aged 6–19 years from Beijing and Zhongshan in China during the period from 2005 to 2018. They observed that each interquartile range increment in PM_2.5_ exposure was significantly associated with a 5.1% increase in the risk of incident overweight or obesity [22]. These findings were similar to our results, which showed that PM_2.5_ had a positive relationship with obesity among children and adolescents in northern and central Taiwan during the period from 2017 to 2022.

Sundram et al. found that long hours of PM_2.5_ exposure affect an individual’s dietary intake, as people with long exposures tend to eat more calories [2]. We assumed that air pollution is a stressful situation for humans; thus, their appetite would tend to increase so that they could gain more energy to face this stressful situation. In addition, Campolim et al. observed that short-term exposure to PM_2.5_ induced hypothalamic inflammation, and long-term exposure led to leptin resistance and obesity in mice [23]. Xu et al. also found that early particulate air pollution exposure had an effect on obesity in mice [24]. In another study, high blood leptin and endothelin-1 levels, vitamin D deficiency, and food reward hormone dysregulation were found in normal weight children exposed to high concentrations of ambient PM_2.5_ in Mexico City [25]. Zordão et al. also found that the leptin, agouti-related peptide, and neuropeptide Y levels in the hypothalamus as well as the food intake were increased in young male mice born from maternal mice with PM_2.5_ exposure during gestation [26]. In Fan et al.’s study, PM_2.5_ and PM_10_ exposures were associated with a significantly higher risk of ADHD [3]. Given that individuals with ADHD had poor impulse control, especially in terms of their appetite, this may explain why air pollution is associated with an increased number of children or adolescents with obesity [27,28].

In our study, we enrolled almost all the children and adolescents who lived in northern and central Taiwan, which allowed us to obtain more accurate estimates. Obesity had a positive correlation with the severity of air pollution with PM_2.5_ or PM_10_. The effect was more significant in girls than in boys, similar to the findings in some studies [29,30]. Guo et al. found that long-term PM_2.5_ exposure attenuated the benefits of physical activity on obesity and that physical activity reduced the risk of obesity among women as compared to their male counterparts [31]. We assumed that long-term PM_2.5_ exposure had a higher positive correlation to obesity among women than among men; thus, the effect of physical activity was more significant among women. Our study results were similar to those of studies conducted in other countries.

The annual average concentrations of PM_2.5_ and PM_10_ should not exceed 5 and 15 mcg/m^3^, respectively, according to the World Health Organization (WHO) Air Quality guidelines for 2021 [32]. However, the Taiwan Air Quality Standard Law announced on 30 September 2024 that the annual average concentrations of PM_2.5_ and PM_10_ should not exceed 12 and 30 mcg/m^3^, respectively [33]. The PM_2.5_ annual average during 2017–2022 in all our studied regions did not meet the PM_2.5_ standard values of the WHO and Taiwan government. Only the cities in Q1 met the PM_10_ standard value of the Taiwan government, but not the WHO standard. Although most of the studied regions met the PM_2.5_ and PM_10_ standard values of the Taiwan government, the top 10% value average could reach twice or even three times the normal standard of the Taiwan government. The abovementioned data indicate that air pollution is still a critical issue in Taiwan.

## 5. Conclusions

The findings of this study underscore the significant impact of PM_2.5_ and PM_10_ exposures on childhood and adolescent obesity, particularly in girls and older students in northern and central Taiwan. PM_2.5_ showed a stronger correlation with obesity than PM_10_, especially during high pollution periods (top 10% annual values). These data highlight the importance of developing and implementing effective measures to reduce air pollution in the region. Additionally, public health policies should prioritize limiting outdoor activities during periods of elevated PM_2.5_ and PM_10_ levels to mitigate associated health risks.

The present study has some limitations. First, given that the government database did not show the socioeconomic status, members of family, food habits, and ethnicity of the studied population, we could not rule out the influences of these biases. Moreover, the pathophysiology between air pollution and obesity remains unclear. Therefore, further research to clarify this question needs to be conducted. Some animal experiments have shown that PM_2.5_ may increase appetite and the serum leptin level. Future research should explore the mechanisms linking air pollution to obesity, such as inflammation, appetite regulation, body fat composition, and gut microbiota, while also developing interventions to mitigate PM_2.5_’s health effects.

## Figures and Tables

**Table 1:** Average PM_2.5_ concentrations during the period from 2017 to 2022, which were categorized into four quartiles. Q1 included cities in the lowest 25% quartile of PM_2.5_ accumulation concentration.

PM_2.5_ Concentration Average During 2017–2022 (μg/m^3^)							
Q1		Shilin	12.72	Q2		Dayuan	14.73
		Zhongzheng	13.32			Pingzhen	14.89
		Wanhua	13.38			Taoyuan	15.39
		Songshan	13.78			Datong	15.40
		Zhongshan	13.83			Shalu	15.57
		Longtan	14.47			Zhongli	15.63
	Average		13.58		Average		15.27
Q3		Guanyin	16.03	Q4		Puli	18.27
		Fengyuan	16.28			Xianxi	18.32
		Dali	16.96			Changhua	18.50
		North (Taichung)	17.81			Erlin	19.36
		Xitun	17.87			Nantou	19.60
					竹山	Zhushan	22.48
	Average		16.99		Average		19.42

**Table 2:** Average PM_10_ concentration during the period from 2017 to 2022, which were categorized into four quartiles. Q1 contained cities in the lowest 25% quartile of PM_10_ accumulation concentration.

PM_10_ Concentration Average During 2017–2022 (μg/m^3^)							
Q1		Zhongzheng	25.20	Q2		Taoyuan	29.43
		Shilin	25.76			Fengyuan	29.57
		Wanhua	26.83			Puli	30.61
		Songshan	27.46			North (Taichung)	31.04
		Zhongshan	28.33			Pingzhen	31.47
	龍潭	Longtan	28.80		大園	Dayuan	32.15
	Average		27.06		Average		30.71
Q3	沙鹿	Shalu	32.56	Q4	大同	Datong	37.08
	中壢	Zhongli	34.29		大里	Dali	37.78
	觀音	Guanyin	35.41		南投	Nantou	38.55
	彰化	Changhua	35.73		線西	Xianxi	39.14
	西屯	Xitun	37.03		竹山	Zhushan	39.99
					二林	Erlin	45.49
	Average		35		Average		39.67

**Table 3:** The number of students in the analysis of PM_2.5_.

The Numbers of Students in PM_2.5_ Statistics				
	Q1	Q2	Q3	Q4
obese boys in elementary school	3687.8	7172.6	3199.5	2424.3
non-obese boys in elementary school	24,500.2	36,905.4	18,209.5	10,486.7
obese girls in elementary school	2129.7	4311.2	2083.6	1531.5
non-obese girls in elementary school	24,026.3	37,270.8	18,461.4	10,357.5
obese boys in junior high school	2027	4797.1	2013.9	1517.4
non-obese boys in junior high school	9602	17,674.9	7976.1	4778.6
obese girls in junior high school	1050.3	2527.3	1386.7	850.2
non-obese girls in junior high school	10,183.7	18,217.7	9937.3	4820.8

**Table 4:** Relative risk of obesity between Q1 and Q2 and between Q3, and Q4 in the analysis of PM_2.5_ accumulation concentration.

		Q2 to Q1	Q3 to Q1	Q4 to Q1
boy in elementary school	RR	1.244	1.142	1.435
	95% CI	1.199–1.29	1.093–1.194	1.369–1.504
girl in elementary school	RR	1.273	1.246	1.582
	95% CI	1.212–1.338	1.176–1.319	1.487–1.683
boy in junior high school	RR	1.225	1.157	1.382
	95% CI	1.169–1.283	1.094–1.223	1.303–1.466
girl in junior high school	RR	1.303	1.31	1.604
	95% CI	1.217–1.395	1.215–1.414	1.474–1.745

**Table 5:** The number of students included in the PM_10_ analysis.

The Numbers of Students in PM_2.5_ Statistics				
	Q1	Q2	Q3	Q4
obese boys in elementary school	3687.8	5477.3	5209.7	2024.5
non-obese boys in elementary school	24,500.2	28,991.7	27,381.3	9313.5
obese girls in elementary school	2129.7	3438.6	3178.9	1220.4
non-obese girls in elementary school	24,026.3	29,339.4	27,464.1	9374.6
obese boys in junior high school	2027	3904.3	2706.8	1709.8
non-obese boys in junior high school	9602	14,548.7	10,144.2	5744.2
obese girls in junior high school	1050.3	2231.2	1470.3	1038.3
non-obese girls in junior high school	10,183.7	16,531.8	10,044.7	6423.7

**Table 6:** Relative risk of obesity between Q1 and Q2, and Q3, and Q4 in the analysis of PM_10_ accumulation concentration.

		Q2 to Q1	Q3 to Q1	Q4 to Q1
boy in elementary school	RR	1.214	1.222	1.365
	95% CI	1.168–1.262	1.175–1.27	1.299–1.434
girl in elementary school	RR	1.288	1.274	1.414
	95% CI	1.224–1.357	1.209–1.342	1.323–1.511
boy in junior high school	RR	1.214	1.208	1.316
	95% CI	1.156–1.274	1.147–1.273	1.243–1.394
girl in junior high school	RR	1.272	1.366	1.488
	95% CI	1.187–1.364	1.267–1.472	1.373–1.613

**Table 7:** PM_2.5_ annual average and annual top 10% average (μg/m^3^).

	2020		2021		2022	
	PM_2.5_	PM_2.5_ Top 10	PM_2.5_	PM_2.5_ Top 10	PM_2.5_	PM_2.5_ Top 10
Shilin	11.91	25.43	12.05	27.22	9.3	19.82
Wanhua	12.31	26.38	12.91	28.83	10.21	21.71
Songshan	13.63	27.7	13.19	29.18	9.58	21.22
Zhongshan	12.96	27.9	13.43	29.47	9.8	21.75
Zhongzheng	11.86	24.99	12.22	27.62	10.69	21.7
Datong	15.94	30.1	15.86	30.7	14.02	24.29
Taoyuan	14.18	31.86	14.52	33.73	12.16	26.08
Dayuan	13.73	32.26	14.03	32.74	11.04	26.36
Guanyin	13.59	31.36	13.74	31.98	12.22	28.08
Pingzhen	13.74	30.1	14.92	32.84	11.53	26.57
Longtan	13.11	29.1	13.05	29.88	8.68	23.24
Zhongli	14.68	31.29	15.48	34.64	12.84	28.17
Fengyuan	14.62	32.12	16.44	34	12.03	27.08
North (Taichung)	15.89	35.3	17.13	37.35	12.45	26.95
Shalu	13.58	33.68	14.33	35.25	12.01	29.76
Dali	16.91	35.97	18.22	40.71	13.51	31.34
Xitun	15.2	34.38	18.31	39.76	13.62	30.74
Changhua	15.18	33.46	17.48	40.57	14	32.15
Xianxi	15.25	34.58	16.87	39.71	14.94	32.26
Erlin	17.37	41.49	19.13	46.35	15.9	37.19
Nantou	17.42	35.76	19.05	38.26	14.93	31.51
Puli	16.6	31.13	16.78	33.38	11.85	25.32
Zhushan	19.53	38.62	20.89	43.93	16.01	36.82

**Table 8:** PM_10_ annual average and annual top 10% average (μg/m^3^).

	2020		2021		2022	
	PM_10_	PM_10_ Top 10	PM_10_	PM_10_ Top 10	PM_10_	PM_10_ Top 10
Shilin	22.89	43.72	24.45	49.08	19.11	36.36
Wanhua	24.2	45.92	23.52	48.51	17.97	35.02
Songshan	24.92	47.68	25.19	50.7	22.92	41.1
Zhongshan	23.86	44.72	24.6	50.23	20.68	38.58
Zhongzheng	21.93	41.3	22.06	45.75	18.52	35.33
Datong	30.41	50.4	31.69	55.91	30.41	48.51
Taoyuan	25.56	51.51	26.46	55.76	21.13	41.24
Dayuan	26.81	54.34	27.7	57.67	23.77	45.9
Guanyin	28.67	56.86	29.48	63.39	25.58	48.53
Pingzhen	26.97	51.94	26.16	54.89	21.05	43.36
Longtan	23.15	46.47	24.02	49.04	18.05	39.32
Zhongli	27.24	51.81	28.63	57.68	26.08	48.43
Fengyuan	26.06	52.18	28.84	55.82	21.74	42.88
North (Taichung)	27.52	58.04	30.56	63.31	24.36	48.02
Shalu	27.03	59.42	31.24	66.16	26.49	53.94
Dali	32.07	64.7	34.87	72.51	29.25	56.69
Xitun	36.12	69.58	33.82	66.05	27.75	53.39
Changhua	29.36	58.85	32.46	65.47	29.08	56.91
Xianxi	36.12	69.58	39.18	80.4	31.07	60.89
Erlin	42.48	81.22	46.02	97.39	38.1	72.1
Nantou	33.14	62.66	33.86	65.51	29.09	53.68
Puli	26.56	46.6	26.77	51.56	22.65	39.97
Zhushan	34.96	64.56	35.91	72.91	29.5	54.89

**Table 9:** Correlation coefficient between obesity or overweight and PM_2.5_ or PM_10_ values in 2020–2022.

2020	OWE Boy	OWE Girl	OBE Boy	OBE Girl	OWJ Boy	OWJ Girl	OBJ Boy	OBJ Girl
PM_2.5_	0.46	0.52	0.54	0.44	0.03	0.18	0.41	0.21
PM_2.5_ top 10	0.55	0.59	0.68	0.61	0.15	0.25	0.64	0.28
PM_10_	0.31	0.46	0.59	0.57	0.23	0.13	0.65	0.29
PM_10_ top 10	0.36	0.47	0.60	0.59	0.09	0.22	0.70	0.27
**2021**	**OWE Boy**	**OWE Girl**	**OBE Boy**	**OBE Girl**	**OWJ Boy**	**OWJ Girl**	**OBJ Boy**	**OBJ Girl**
PM_2.5_	0.18	0.39	0.54	0.44	0.12	0.20	0.38	0.35
PM_2.5_ top 10	0.29	0.43	0.62	0.56	0.09	0.09	0.55	0.41
PM_10_	0.18	0.33	0.64	0.60	0.26	−0.05	0.66	0.52
PM_10_ top 10	0.28	0.38	0.67	0.65	0.23	−0.10	0.72	0.53
**2022**	**OWE boy**	**OWE Girl**	**OBE Boy**	**OBE Girl**	**OWJ Boy**	**OWJ Girl**	**OBJ Boy**	**OBJ Girl**
PM_2.5_	0.46	0.44	0.60	0.56	0.10	0.22	0.62	0.45
PM_2.5_ top 10	0.56	0.62	0.74	0.70	−0.07	0.25	0.70	0.54
PM_10_	0.37	0.36	0.54	0.52	0.19	0.04	0.65	0.40
PM_10_ top 10	0.41	0.40	0.59	0.60	0.11	0.08	0.70	0.45

OWE: overweight, elementary school; OBE: obese, elementary school; OWJ: overweight junior high school; OBJ: obese, junior high school.

## Data Availability

The data that support the findings of this study are available from K-12 Education Administration, Ministry of Education, R.O.C. (Taiwan). Restrictions apply to the availability of these data, which were used under license for this study. Data are available with the permission of K-12 Education Administration, Ministry of Education, R.O.C. (Taiwan).

## References

[1] Tamayo-Ortiz M., Téllez-Rojo M.M., Rothenberg S.J., Gutiérrez-Avila I., Just A.C., Kloog I., Texcalac-Sangrador J.L., Romero-Martinez M., Bautista-Arredondo L.F., Schwartz J. (2021). Exposure to PM_2.5_ and Obesity Prevalence in the Greater Mexico City Area. Int. J. Environ. Res. Public Health.

[2] Sundram T.K.M., Tan E.S.S., Lim H.S., Amini F., Bustami N.A., Tan P.Y., Rehman N., Bin Ho Y., Tan C.K. (2022). Effects of Ambient Particulate Matter (PM_2.5_) Exposure on Calorie Intake and Appetite of Outdoor Workers. Nutrients.

[3] Fan H.-C., Chen C.-M., Tsai J.-D., Chiang K.-L., Tsai S.C.-S., Huang C.-Y., Lin C.-L., Hsu C.Y., Chang K.-H. (2022). Association between Exposure to Particulate Matter Air Pollution during Early Childhood and Risk of Attention-Deficit/Hyperactivity Disorder in Taiwan. Int. J. Environ. Res. Public Health.

[4] Li S., Wu W., Wang G., Zhang X., Guo Q., Wang B., Cao S., Yan M., Pan X., Xue T. (2022). Association between exposure to air pollution and risk of allergic rhinitis: A systematic review and meta-analysis. Environ Res..

[5] Paciência I., Rufo J.C., Moreira A. (2022). Environmental inequality: Air pollution and asthma in children. Pediatr. Allergy Immunol..

[6] Zou M.-L., Jiang C.-B., Chen Y.-H., Wu C.-D., Lung S.-C.C., Chien L.-C., Kallawicha K., Lo Y.-C., Chao H.J. (2022). Frequent occurrence of respiratory symptoms in children is associated with exposure to air pollution, land use types, and parental mental health in the Greater Taipei area. Environ. Res..

[7] Zhang S., Qian Z.M., Chen L., Zhao X., Cai M., Wang C., Zou H., Wu Y., Zhang Z., Li H. (2023). Exposure to Air Pollution during Pre-Hypertension and Subsequent Hypertension, Cardiovascular Disease, and Death: A Trajectory Analysis of the UK Biobank Cohort. Environ. Health Perspect..

[8] Zhang J.-S., Gui Z.-H., Zou Z.-Y., Yang B.-Y., Ma J., Jing J., Wang H.-J., Luo J.-Y., Zhang X., Luo C.-Y. (2021). Long-term exposure to ambient air pollution and metabolic syndrome in children and adolescents: A national cross-sectional study in China. Environ. Int..

[9] Leung A.K.C., Wong A.H., Hon K.L. (2024). Childhood Obesity: An Updated Review. Curr. Pediatr. Rev..

[10] Shi L., Jiang Z., Zhang L. (2022). Childhood obesity and central precocious puberty. Front. Endocrinol..

[11] Styne D.M., Arslanian S.A., Connor E.L., Farooqi I.S., Murad M.H., Silverstein J.H., Yanovski J.A. (2017). Pediatric Obesity—Assessment, Treatment, and Prevention: An Endocrine Society Clinical Practice Guideline. J. Clin. Endocrinol. Metab..

[12] Parasin N., Amnuaylojaroen T., Saokaew S. (2021). Effect of Air Pollution on Obesity in Children: A Systematic Review and Meta-Analysis. Children.

[13] Zhang Z., Dong B., Chen G., Song Y., Li S., Yang Z., Dong Y., Wang Z., Ma J., Guo Y. (2021). Ambient air pollution and obesity in school-aged children and adolescents: A multicenter study in China. Sci. Total Environ..

[14] Zheng J., Zhang H., Shi J., Li X., Zhang J., Zhang K., Gao Y., He J., Dai J., Wang J. (2024). Association of air pollution exposure with overweight or obesity in children and adolescents: A systematic review and meta–analysis. Sci. Total Environ..

[15] School Basic Statistics Information [Internet]. Department of Statistics, Ministry of Education, R.O.C. (Taiwan). https://depart.moe.edu.tw/ED4500/News_Content.aspx?n=5A930C32CC6C3818&sms=91B3AAE8C6388B96&s=4F9035F0AF08D527.

[16] Taiwan Air Quality Monitoring Network [Internet]. Ministry of Environment, R.O.C. (Taiwan). https://airtw.moenv.gov.tw/CHT/Query/His_Data.aspx.

[17] Chen W., Chang M.-H. (2010). New growth charts for Taiwanese children and adolescents based on World Health Organization standards and health-related physical fitness. Pediatr. Neonatol..

[18] Chen C.-H., Huang L.-Y., Lee K.-Y., Wu C.-D., Chiang H.-C., Chen B.-Y., Chin W.-S., Pan S.-C., Guo Y.L. (2019). Effects of PM_2.5_ on Skeletal Muscle Mass and Body Fat Mass of the Elderly in Taipei, Taiwan. Sci. Rep..

[19] Luo C., Wei T., Jiang W., Yang Y.-P., Zhang M.-X., Xiong C.-L., Tung T.-H. (2024). The association between air pollution and obesity: An umbrella review of meta-analyses and systematic reviews. BMC Public Health.

[20] Huang S., Zhang X., Liu Z., Liang F., Li J., Huang K., Yang X., Chen J., Liu X., Cao J. (2021). Long-term impacts of ambient fine particulate matter exposure on overweight or obesity in Chinese adults: The China-PAR project. Environ. Res..

[21] Tong J., Ren Y., Liu F., Liang F., Tang X., Huang D., An X., Liang X. (2022). The Impact of PM_2.5_ on the Growth Curves of Children’s Obesity Indexes: A Prospective Cohort Study. Front. Public Health.

[22] Wang Y., Li W., Chen S., Zhang J., Liu X., Jiang J., Chen L., Tang Z., Wan X., Lian X. (2024). PM_2.5_ constituents associated with childhood obesity and larger BMI growth trajectory: A 14-year longitudinal study. Environ. Int..

[23] Campolim C.M., Weissmann L., Ferreira C.K.d.O., Zordão O.P., Dornellas A.P.S., de Castro G., Zanotto T.M., Boico V.F., Quaresma P.G.F., Lima R.P.A. (2020). Short-term exposure to air pollution (PM_2.5_) induces hypothalamic inflammation, and long-term leads to leptin resistance and obesity via Tlr4/Ikbke in mice. Sci. Rep..

[24] Xu X., Yavar Z., Verdin M., Ying Z., Mihai G., Kampfrath T., Wang A., Zhong M., Lippmann M., Chen L.-C. (2010). Effect of early particulate air pollution exposure on obesity in mice: Role of p47phox. Arter. Thromb. Vasc. Biol..

[25] Calderón-Garcidueñas L., Franco-Lira M., D’Angiulli A., Rodríguez-Díaz J., Blaurock-Busch E., Busch Y., Chao C.-K., Thompson C., Mukherjee P.S., Torres-Jardón R. (2015). Mexico City normal weight children exposed to high concentrations of ambient PM_2.5_ show high blood leptin and endothelin-1, vitamin D deficiency, and food reward hormone dysregulation versus low pollution controls. Relevance for obesity and Alzheimer disease. Environ. Res..

[26] Zordão O.P., Campolim C.M., Yariwake V.Y., Castro G., Ferreira C.K.d.O., Santos A., Norberto S., Veras M.M., Saad M.J.A., Saldiva P.H.N. (2023). Maternal exposure to air pollution alters energy balance transiently according to gender and changes gut microbiota. Front. Endocrinol..

[27] Karhunen V., Bond T.A., Zuber V., Hurtig T., Moilanen I., Järvelin M.-R., Evangelou M., Rodriguez A. (2021). The link between attention deficit hyperactivity disorder (ADHD) symptoms and obesity-related traits: Genetic and prenatal explanations. Transl. Psychiatry.

[28] Cortese S. (2019). The Association between ADHD and Obesity: Intriguing, Progressively More Investigated, but Still Puzzling. Brain Sci..

[29] Li M., Qian Z., Vaughn M., Boutwell B., Ward P., Lu T., Lin S., Zhao Y., Zeng X.-W., Liu R.-Q. (2015). Sex-specific difference of the association between ambient air pollution and the prevalence of obesity in Chinese adults from a high pollution range area: 33 Communities Chinese Health Study. Atmos. Environ..

[30] Liu X., Tu R., Qiao D., Niu M., Li R., Mao Z., Huo W., Chen G., Xiang H., Guo Y. (2020). Association between long-term exposure to ambient air pollution and obesity in a Chinese rural population: The Henan Rural Cohort Study. Environ. Pollut..

[31] Guo Q., Xue T., Wang B., Cao S., Wang L., Zhang J., Duan X. (2022). Effects of physical activity intensity on adulthood obesity as a function of long-term exposure to ambient PM2.5: Observations from a Chinese nationwide representative sample. Sci. Total Environ..

[32] WHO Global Air Quality Guidelines, 22 September 2021. https://www.who.int/publications/i/item/9789240034228.

[33] Air Quality Standards Regulations. https://airtw.moenv.gov.tw/CHT/Information/Standard/Rules.aspx.

